# RETRACTION: Quality of Evidence Supporting the Role of Hyperbaric Oxygen Therapy for Diabetic Foot Ulcers

**DOI:** 10.1111/iwj.70606

**Published:** 2025-04-21

## Abstract

**RETRACTION:** JiangF. , ZhangY. , ChengS. , YangX. , BaiM. , and ZhangM. , “Quality of Evidence Supporting the Role of Hyperbaric Oxygen Therapy for Diabetic Foot Ulcers,” International Wound Journal 20, no. 4 (2024): e14530, 10.1111/iwj.14530.PMC1096103038053520

The above article, published online on 06 December 2023, in Wiley Online Library (http://onlinelibrary.wiley.com/), has been retracted by agreement between the journal Editor in Chief, Professor Keith Harding; and John Wiley & Sons, Ltd. Following an investigation by the publisher, all parties have concluded that this article was accepted solely on the basis of a compromised peer review process. The editors have therefore decided to retract the article. The authors did not respond to our notice regarding the retraction.

**RETRACTION:** F. Jiang , Y. Zhang , S. Cheng , X. Yang , M. Bai , and M. Zhang , “Quality of Evidence Supporting the Role of Hyperbaric Oxygen Therapy for Diabetic Foot Ulcers,” International Wound Journal 20, no. 4 (2024): e14530, 10.1111/iwj.14530.PMC1096103038053520

The above article, published online on 06 December 2023, in Wiley Online Library (http://onlinelibrary.wiley.com/), has been retracted by agreement between the journal Editor in Chief, Professor Keith Harding; and John Wiley & Sons, Ltd. Following an investigation by the publisher, all parties have concluded that this article was accepted solely on the basis of a compromised peer review process. The editors have therefore decided to retract the article. The authors did not respond to our notice regarding the retraction.

